# Long non-coding RNA PVT1 promotes osteosarcoma development by acting as a molecular sponge to regulate miR-195

**DOI:** 10.18632/oncotarget.13012

**Published:** 2016-11-02

**Authors:** Quan Zhou, Fengli Chen, Jiali Zhao, Baojun Li, Yong Liang, Wei Pan, Shaoxian Zhang, Xinhong Wang, Donghui Zheng

**Affiliations:** ^1^ Department of Orthopaedics, Huai'an Hospital Affiliated of Xuzhou Medical University and Huai'an Second Hospital, Huai'an 223002, Jiangsu, China; ^2^ Department of Central Laboratory, Huai'an First People's Hospital, Nanjing Medical University, Huai'an 223002, Jiangsu, China; ^3^ Department of Central Laboratory and Department of Nephrology, Huai'an Hospital Affiliated of Xuzhou Medical University and Huai'an Second Hospital, Huai'an 223002, Jiangsu, China; ^4^ Department of Joint Surgery, Second People's Hospital of Hunan Province, Changsha 410007, Hunan, China

**Keywords:** PVT1, miR-195, osteosarcoma, proliferation, invasion

## Abstract

A growing body of evidence has indicated that long non-coding RNAs (lncRNAs) serve as competing endogenous RNAs (ceRNAs) during oncogenesis. In this study, the qRT-PCR results indicated that the lncRNA PVT1 is overexpressed in osteosarcoma and decreased the survival rate of osteosarcoma patients. MTT and clonal colony formation assays were used to detect the effect of PVT1 on proliferation, and flow cytometry was performed to assess apoptosis and the cell cycle. A Transwell assay was used to analyze migration and invasion. The results revealed that silencing PVT1 by siRNA inhibited proliferation, migration and invasion and promoted apoptosis and cell cycle arrest in osteosarcoma cells. Furthermore, a gene microarray was used to screen differentially expressed miRNAs associated with PVT1. The interaction between PVT1 and miRNAs was then analyzed by qRT-PCR and luciferase reporter gene assay. We found that PVT1 negatively regulated miR-195 in osteosarcoma cells. Simultaneously, we found that silencing PVT1 by siRNA suppressed proliferation, migration and invasion and promoted cell cycle arrest and apoptosis via miR-195 in osteosarcoma cells. Moreover, silencing PVT1 by siRNA inhibited BCL2, CCND1, and FASN protein expression via miR-195 in osteosarcoma cells, and BCL2 inhibited the si-PVT1#1-induced apoptosis of U2OS cells. CCND1 inhibited the cell cycle arrest of U2OS cells induced by si-PVT1#1. FASN promoted the invasiveness U2OS cells, which was inhibited by si-PVT1#1. Therefore, our study demonstrated that PVT1 may be a therapeutic target for treatment of osteosarcoma.

## INTRODUCTION

Osteosarcoma is a malignant primary bone tumor that is lethal to primarily children and adolescents [1, 2]. The incidence of osteosarcoma is moderate, approximately 10 to 26 new cases per million worldwide annually [3]. Locally, osteosarcoma is highly aggressive and rapidly metastasizes, which results in poor survival [4]. Specifically, the 5-year survival rate of osteosarcoma patients, most of whom died of lung metastases, was less than 20% in the first half of the 20th century [4, 5]. At present, surgery, chemotherapy, and palliative radiotherapy are the main treatment methods for patients with osteosarcoma. However, the efficacy of conventional therapy is limited, and osteosarcoma diagnosis is restricted because the molecular and functional mechanisms of this disease are poorly understood. Therefore, further research is needed to identify effective biomarkers and molecular targets.

Most RNAs that are transcribed are long non-coding RNAs (lncRNAs), which are longer than 200 nucleotides. An increasing number of studies have revealed that lncRNAs participate in the cell cycle, cell differentiation [6], and apoptosis [7, 8], and LncRNAs are now regarded as new regulatory genes that contribute to cancer development and progression [9]. Specifically, LncRNAs may play critical roles in the formation and development of cancers by suppressing or promoting tumors [10]. At present, studies have demonstrated that lncRNAs are involved in a variety of tumors, such as such as liver [11], lung [12], and breast cancer [13–15]. Therefore, lncRNAs are considered important therapeutic targets for diseases, but the mechanism and function of the lncRNA PVT1 in osteosarcoma are poorly understood.

MicroRNAs (miRNAs), which are 19–22 nucleotides long, regulate gene expression by targeting the 3′-UTR of target genes [16]. Specifically, they participate in the development of various diseases by affecting biological processes, such as differentiation, the cell cycle, and apoptosis [17–20], and an increasing number of studies have proven that miRNAs play vital roles in cancer by regulating genes, including deletion, amplification, mutation, and epigenetic silencing [21]. Therefore, miRNAs may serve as new and effective biomarkers for human cancer diagnostics.

Studies have also indicated that lncRNAs serve as competitive endogenous RNAs (ceRNAs) and are involved in the occurrence and development of various diseases [22–24]. For example, lncRNAs acting as ceRNAs play vital roles in pulmonary fibrosis [25]; LncRNA H19 acts as an miRNA sponge to accelerate epithelial to mesenchymal transition (EMT) in colorectal cancer [26]. LncRNA-H19 also acts on miR-141 to regulate the proliferation and migration of gastric cancer cells [27]. Furthermore, LncRNA-MEG3 acts as a ceRNA to regulate gastric cancer progression [28]. In our study, we show that PVT1 negatively regulates miR-195 transcription.

Specifically, the current study indicates the effects of PVT1 in osteosarcoma, the functional mechanisms of the interaction between PVT1 and miRNAs, and the roles of potential downstream genes. Our research also demonstrates that PVT1 may be a therapeutic target of osteosarcoma.

## RESULTS

### LncRNA PVT1 is overexpressed in osteosarcoma and decreases the survival rate of osteosarcoma patients

**Figure 1:**
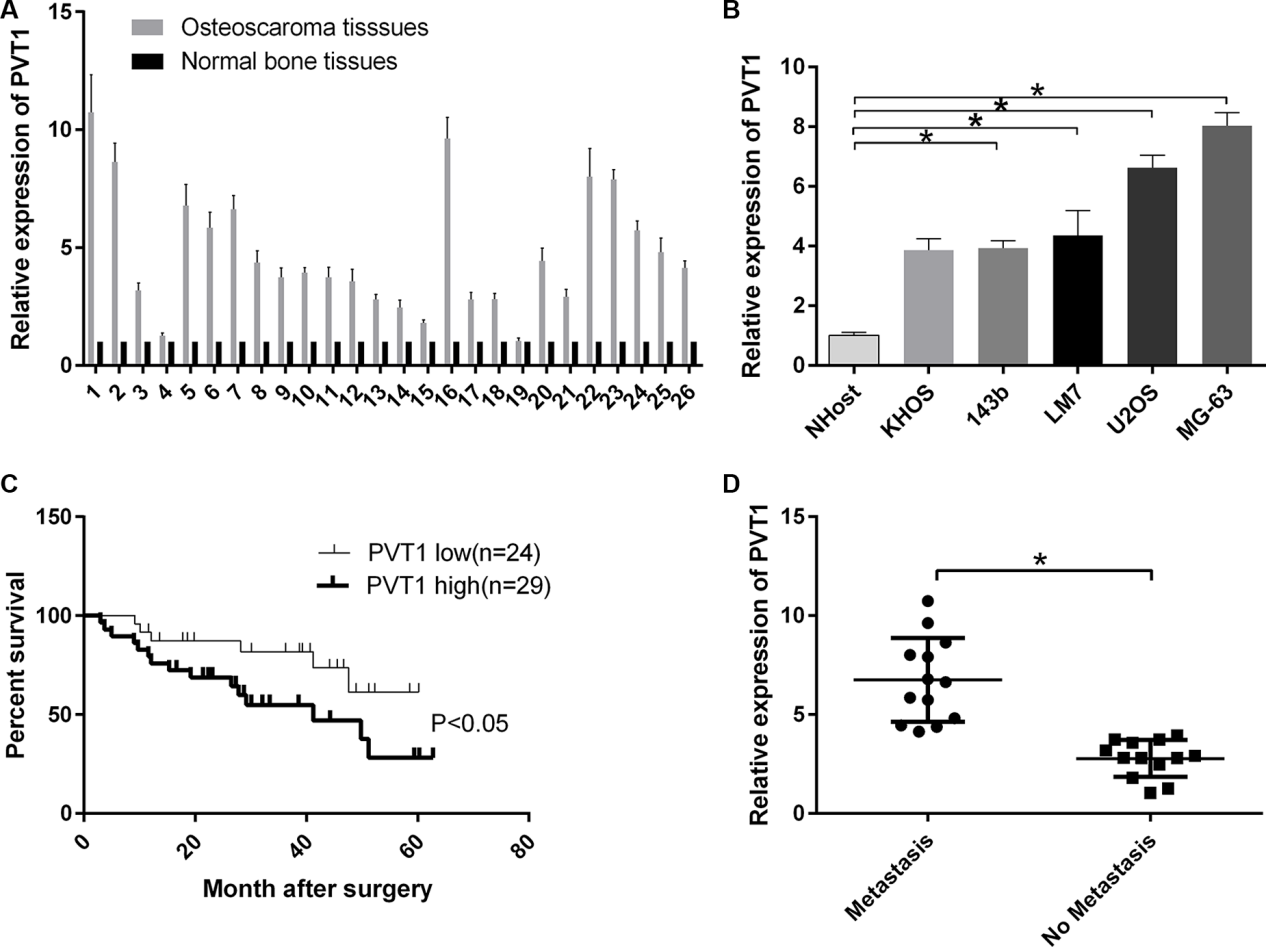
LncRNA PVT1 is overexpressed in osteosarcoma and decreases the survival rate of osteosarcoma patients (**A**) The mRNA expression level of PVT1 was measured by qRT-PCR in osteosarcoma tissues (*n* = 26) and corresponding noncancerous tissues (*n* = 26). (**B**) The mRNA expression level of PVT1 was measured by qRT-PCR in the normal osteoblast cell line NHost and various osteosarcoma cell lines (KHOS, 143b, LM7, U2OS, and MG-63) (^*^P < 0.05). (**C**) Comparison of survival curves between tumors expressing high levels of PVT1 (*n* =29) and tumors expressing low levels of PVT (*n* = 24). (**D**) qRT-PCR was used to measure the mRNA expression level of PVT1 in metastatic osteosarcoma tissues (*n* = 13) and primary osteosarcoma tissues (*n* = 13).

We randomly collected osteosarcoma tissues and corresponding noncancerous tissues from 26 patients. The mRNA expression level of PVT1 was measured by qRT-PCR and increased in osteosarcoma tissues compared with corresponding noncancerous tissues (Figure 1A). Moreover, we found that the mRNA expression level of PVT1 was upregulated in osteosarcoma cell lines (KHOS, 143b, LM7, U2OS, and MG-63) compared with a normal osteoblast cell line NHost (*P* < 0.05), and PVT1 expression was higher in U2OS and MG-63 cells than other osteosarcoma cells (Figure 1B). The results also indicated that PVT1 reduced the survival rate of osteosarcoma patients (*P* < 0.05) (Figure 1C). Furthermore, the results showed that the mRNA expression level of PVT1 was higher in metastatic osteosarcoma tissues than primary osteosarcoma tissues (*P* < 0.05) (Figure 1D).

### Silencing PVT1 by siRNA inhibits proliferation and promotes apoptosis in osteosarcoma cells

**Figure 2:**
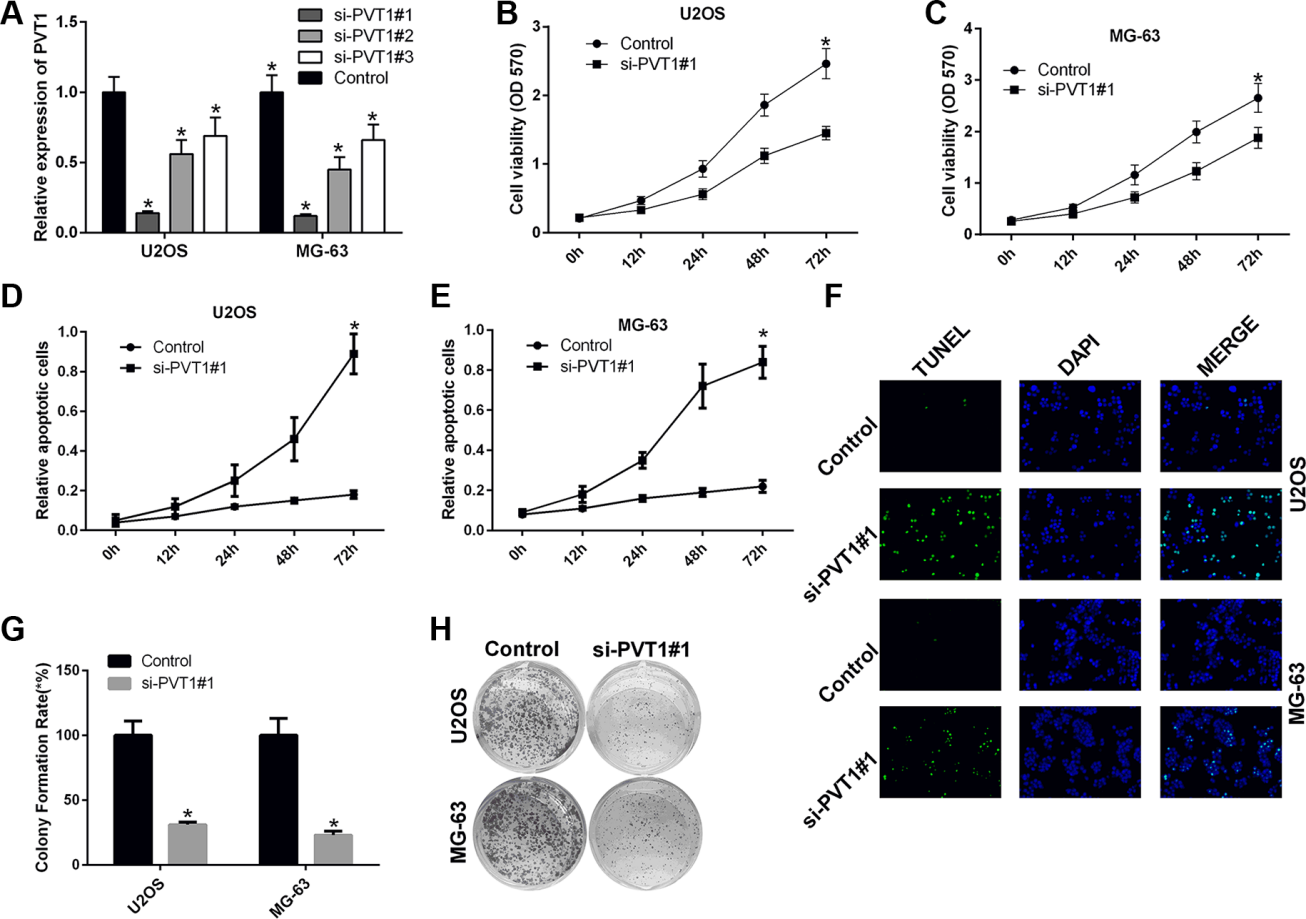
Silencing PVT1 by siRNA inhibits proliferation and promotes apoptosis in osteosarcoma cells (**A**) qRT-PCR was used to measure the expression level of PVT1 in U2OS and MG-63 cells that had been transfected with control or siRNAs against PVT1, i.e., si-PVT1#1, si-PVT1#2, or si-PVT1#3 (^*^P < 0.05). (**B**–**C**) Cell proliferation was assessed with an MTT assay in U2OS and MG-63 cells transfected with control or si-PVT1#1 (^*^P < 0.05). (**D**–**E**) Cell apoptosis was assessed by Annexin-V/7-AAD staining in U2OS and MG-63 cells transfected with control or si-PVT1#1 (^*^P < 0.05). (**F**) An immunofluorescence assay was performed to measure TUNEL expression in U2OS and MG-63 cells transfected with control or si-PVT1#1. (**G**–**H**) A clonal colony-forming assay was performed to assess cell proliferation in U2OS and MG-63 cells transfected with control or si-PVT1#1 (^*^P < 0.05).

We also studied the impact of silencing PVT1 on the proliferation and apoptosis of osteosarcoma cells. To this end, U2OS and MG-63 cells were transfected with control siRNA or siRNAs against PVT1, i.e., si-PVT1#1, si-PVT1#2, and si-PVT1#3. The qRT-PCR results indicated that si-PVT1#1 effectively knocked down PVT1 (Figure 2A). Thus, U2OS and MG-63 cells were transfected with control or si-PVT1#1. The MTT results showed that silencing PVT1 by siRNA inhibited the proliferation of U2OS and MG-63 cells (*P* < 0.05) (Figure 2B–2C). The apoptosis assay results indicated that silencing PVT1 by siRNA induced the apoptosis of U2OS and MG-63 cells (*P* < 0.05) (Figure 2D–2E). As previously described [29], terminal dUTP nick-end labeling (TUNEL) can be used to detect late-stage apoptosis based on the detection of fragmented DNA. The immunofluorescence results further proved that silencing PVT1 by siRNA induced U2OS and MG-63 cell apoptosis (Figure 2F). Moreover, the clonal colony forming assay results also showed that silencing PVT1 by siRNA inhibited the proliferation of U2OS and MG-63 cells (*P* < 0.05) (Figure 2G–2H).

### Silencing PVT1 by siRNA inhibits migration and invasion and induces cell cycle arrest in osteosarcoma cells

**Figure 3:**
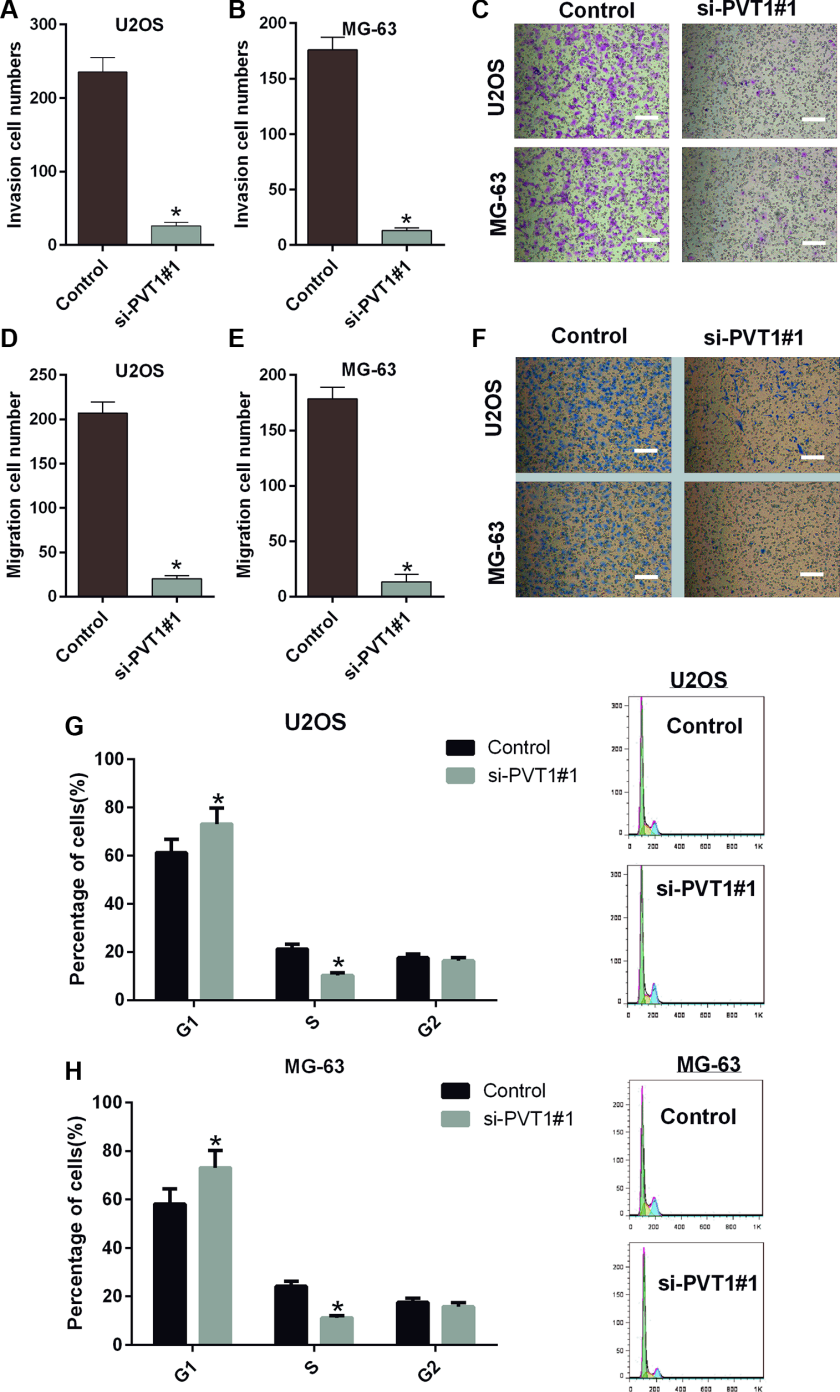
Silencing PVT1 by siRNA inhibits migration and invasion and induces cell cycle arrest in osteosarcoma cells (**A**–**C**) A Transwell assay was performed to assess the migration of U2OS and MG-63 cells transfected with control or si-PVT1#1; Magnification 200×, Scale bars = 10 μm, (^*^P < 0.05). (**D**–**F**) A Transwell assay was performed to assess the invasiveness of U2OS and MG-63 cells transfected with control or si-PVT1#1; Magnification 200×, Scale bars = 10 μm, (^*^P < 0.05). (**G**) The cell cycle distribution was assessed by flow cytometry in U2OS cells transfected with control or si-PVT1#1. (H) The cell cycle distribution was detected by flow cytometry in MG-63 cells transfected with control or si-PVT1#1.

We also studied the impacts of silencing PVT1 on the migration, invasion and cell cycle of osteosarcoma cells. Similarly, U2OS and MG-63 cells were transfected with control or si-PVT1#1. Our results indicated that the migration capacities of U2OS and MG-63 cells transfected with si-PVT1#1 were significantly decreased compared with the control group (*P* < 0.05) (Figure 3A–3C). The invasion capacities of U2OS and MG-63 cells transfected with si-PVT1#1 were also significantly decreased compared with the control group (*P* < 0.05) (Figure 3D–3F). Furthermore, PVT1 silencing-induced cell cycle arrest in U2OS cells was assessed by flow cytometry. We found a significant increase in the number of G1-phase cells in U2OS transfected with si-PVT1#1 compared with control cells and a significant decrease in S-phase cells in U2OS cells transfected with si-PVT1#1 compared with control, indicating that the growth-inhibiting effect of silencing PVT1 was due to arrest at the G1-S phase transition (Figure 3G). A similar result was found in MG-63 cells (Figure 3H).

### PVT1 negatively regulates miR-195 in osteosarcoma cells

**Figure 4:**
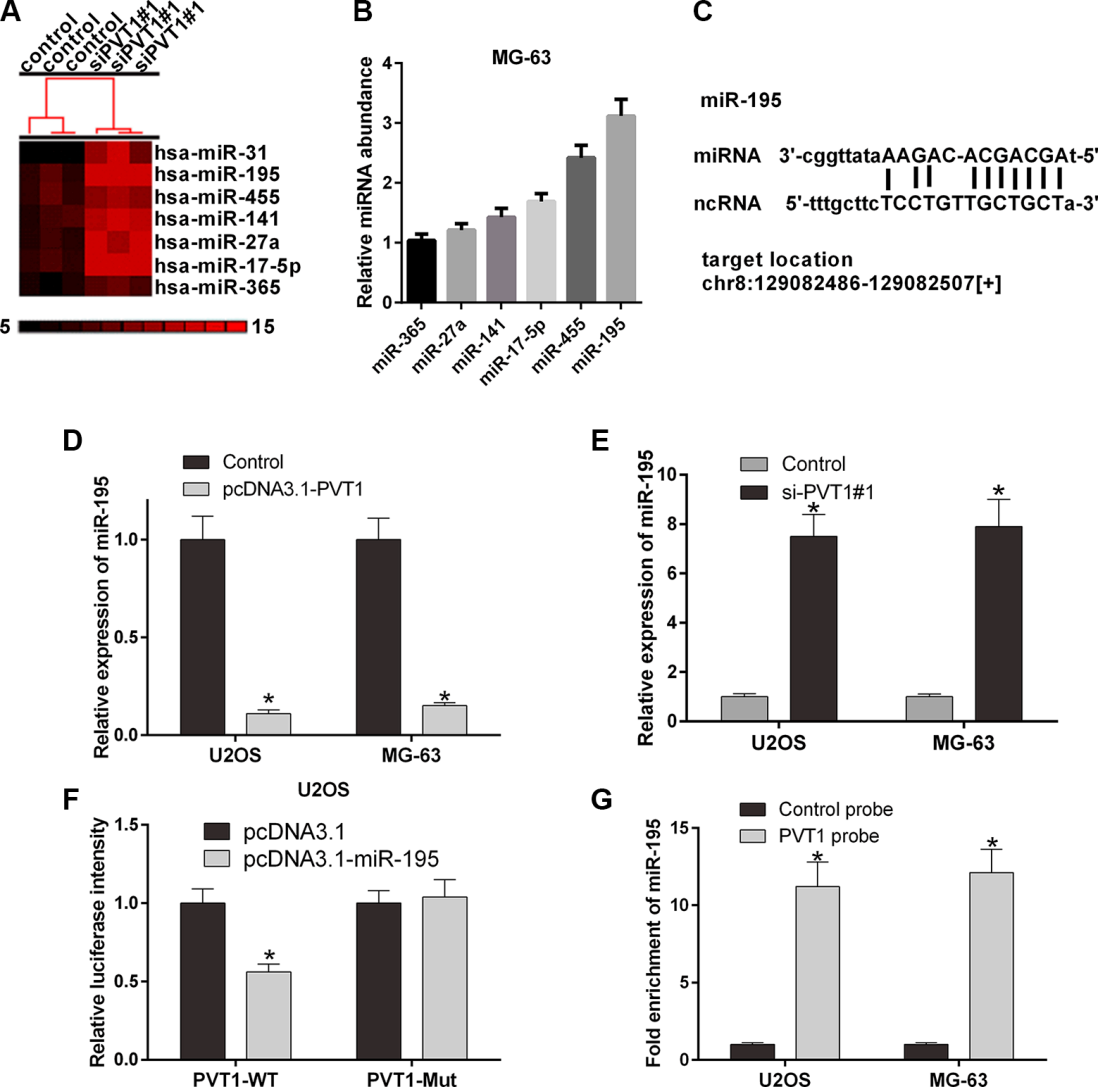
PVT1 negatively regulates miR-195 in osteosarcoma cells (**A**) The cluster heat map of differentially expressed miRNAs in U2OS cells transfected with control or si-PVT1#1 on a scale from black (low) to red (high). Individual cell subsets are depicted as columns. (**B**) qRT-PCR was used to measure the mRNA expression level of PVT1 in MG-63 cells transfected with the predicted miRNAs, i.e., miR-365, miR-27a, miR-141, miR-17-5p, miR-455, or miR-195. (**C**) The putative binding site of miR-195 and LncRNA PVT1 is shown. (**D**) The mRNA expression level of miR-195 was analyzed by qRT-PCR in U2OS and MG-63 cells transfected with control or pcDNA3.1- PVT1 (^*^P < 0.05). (**E**) The mRNA expression level of miR-195 was analyzed by qRT-PCR in U2OS and MG-63 cells transfected with control or si-PVT1#1 (^*^P < 0.05). (**F**) The luciferase activity of PVT1 was detected using the luciferase report gene assay in U2OS cells co-transfected the wild type (PVT1-WT) or mutant (PVT1-Mut) and pcDNA3.1 or pcDNA3.1-miR-195 (*P < 0.05). (**G**) miR-195 was significantly enriched by a chromatin Immunoprecipitation (ChIP) assay in U2OS and MG-63 cells transfected with control or PVT1 probe (*P < 0.05).

To investigate the interaction between PVT1 and miRNAs, we predicted the miRNAs that may interact with PVT1 using starBase v2.0 (Table 1). U2OS and MG-63 cells were then transfected with control siRNA or si-PVT1#1. Furthermore, a gene microarray was used to screen differentially expressed miRNAs associated with PVT1. We selected 7 miRNAs, miR-365, miR-27a, miR-141, miR-17-5p, miR-455, and miR-195 (Figure 4A). qRT-PCR was performed to validate the mRNA expression levels of miRNAs in MG-63 cells. Our results revealed that miR-195 was highly expressed in MG-63 cells (Figure 4B). We then studied the interaction between PVT1 and miR-195. The results indicated that the overexpression of PVT1 decreased the mRNA expression level of miR-195 (Figure 4D), whereas silencing PVT1 increased the mRNA expression level of miR-195 (Figure 4E). The PVT1 sequence and the predicted binding site of miR-195 were then analyzed (Figure 4C), and the PVT1 sequence was cloned into the region downstream of the luciferase gene. The results indicated that the luciferase activity of PVT1 was significantly decreased in U2OS cells co-transfected the wild type (PVT1-WT) and miR-195 compared with mutant (PVT1-Mut) and miR-195 (*P* < 0.05) (Figure 4F). Moreover, miR-195 was significantly enriched by PVT1 in U2OS and MG-63 cells (*P* < 0.05) (Figure 4G).

**Table 1:** The interaction between PVT1 and miRNAs were predicted by starBase v2.0

miRNA name	mirAccession	geneName	targetSites
hsa-miR-186-5p	MIMAT0000456	PVT1	1
hsa-miR-190b	MIMAT0004929	PVT1	1
hsa-miR-488-3p	MIMAT0004763	PVT1	1
hsa-miR-16-5p	MIMAT0000069	PVT1	1
hsa-miR-15a-5p	MIMAT0000068	PVT1	1
hsa-miR-17-5p	MIMAT0000070	PVT1	2
hsa-miR-20a-5p	MIMAT0000075	PVT1	2
hsa-miR-544a	MIMAT0003164	PVT1	1
hsa-miR-203a	MIMAT0000264	PVT1	1
hsa-miR-190a-5p	MIMAT0000458	PVT1	1
hsa-miR-365a-3p	MIMAT0000710	PVT1	1
hsa-miR-195-5p	MIMAT0000461	PVT1	1
hsa-miR-497-5p	MIMAT0002820	PVT1	1
hsa-miR-24-3p	MIMAT0000080	PVT1	1
hsa-miR-519d-3p	MIMAT0002853	PVT1	2
hsa-miR-128-3p	MIMAT0000424	PVT1	1
hsa-miR-15b-5p	MIMAT0000417	PVT1	1
hsa-miR-93-5p	MIMAT0000093	PVT1	2
hsa-miR-106b-5p	MIMAT0000680	PVT1	2
hsa-miR-455-5p	MIMAT0003150	PVT1	1
hsa-miR-20b-5p	MIMAT0001413	PVT1	2
hsa-miR-106a-5p	MIMAT0000103	PVT1	2
hsa-miR-424-5p	MIMAT0001341	PVT1	1

### Silencing PVT1 by siRNA suppresses proliferation, migration and invasion and promotes cell cycle arrest and apoptosis via miR-195 in osteosarcoma cells

**Figure 5:**
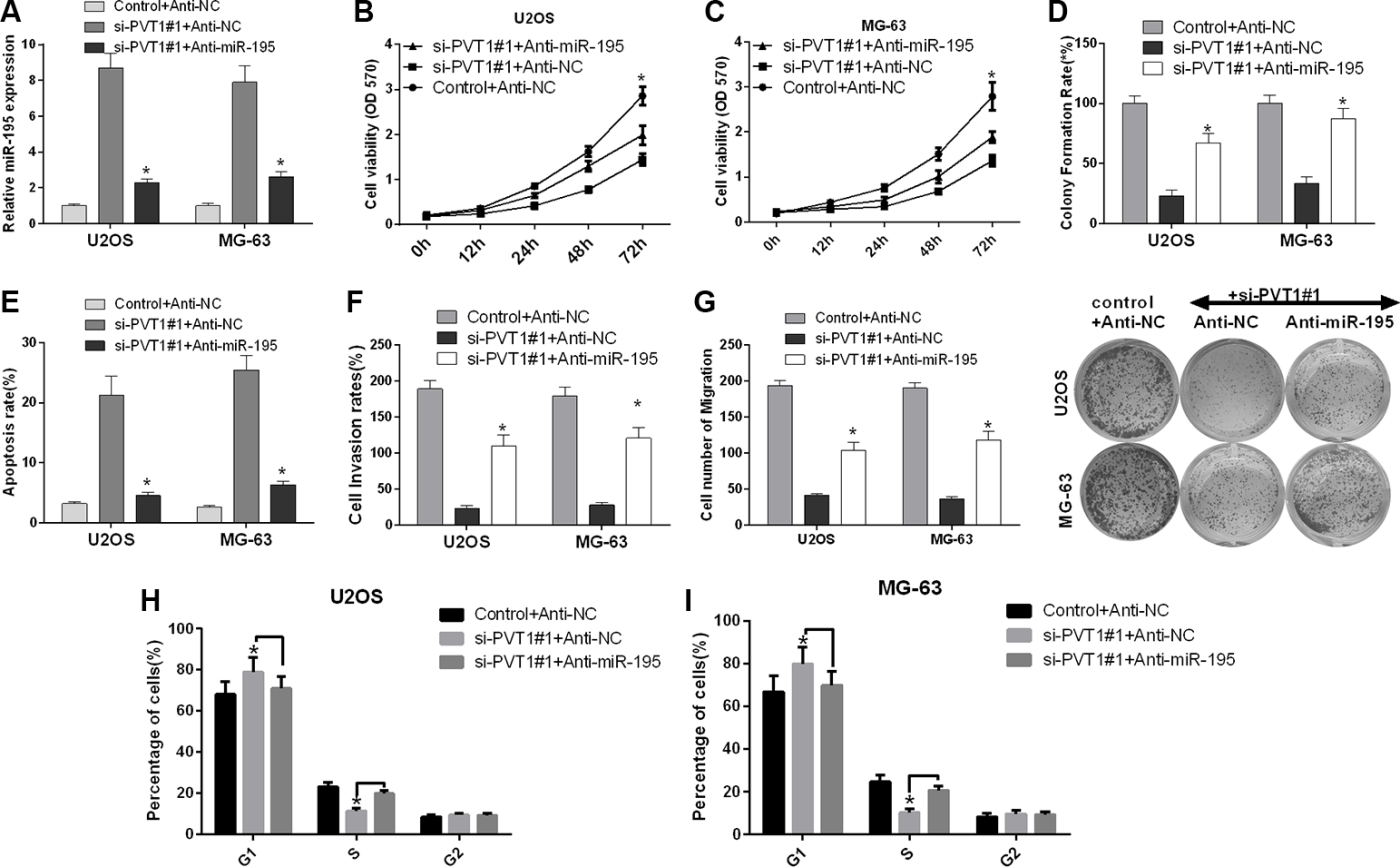
Silencing PVT1 by siRNA suppresses proliferation, migration and invasion and promotes cell cycle arrest and apoptosis via miR-195 in osteosarcoma cells (**A**) U2OS and MG-63 cells were transfected with control and anti-NC, si-PVT1#1 and anti-NC, or si-PVT1#1 and anti-miR-195. The mRNA expression level of miR-195 was detected by qRT-PCR (^*^P < 0.05). (**B**–**C**) Cell proliferation was assessed with an MTT assay in U2OS and MG-63 cells treated as described in A (^*^P < 0.05). (**D**) A clonal colony-forming assay was performed to assess the proliferation of U2OS and MG-63 cells treated as described in A (^*^P < 0.05). (**E**) Apoptosis was assessed by Annexin-V/7-AAD staining in U2OS and MG-63 cells treated as described in A (^*^P < 0.05). (F) The invasion of U2OS and MG-63 cells treated as described in A was assessed using a Transwell assay (^*^P < 0.05). (**G**) The migration of U2OS and MG-63 cells treated as described in A was assessed using a Transwell assay (^*^P < 0.05). (**H**–**I**) The cell cycle distribution was analyzed by flow cytometry in U2OS and MG-63 cells treated as described in A.

We further investigated the role of miR-195 in the influence of PVT1 expression on the proliferation, cell cycle, apoptosis, migration and invasion of osteosarcoma cells. U2OS and MG-63 cells were transfected with control siRNA and anti-NC, si-PVT1#1 and anti-NC, or si-PVT1#1 and anti-miR-195. qRT-PCR was used to detect the mRNA expression level of miR-195. Our results revealed that miR-195 was up-regulated in U2OS and MG-63 cells transfected with si-PVT1#1 compared with control cells, and miR-195 was down-regulated in U2OS and MG-63 cells transfected with si-PVT1#1 and anti-miR-195 compared with cells transfected with si-PVT1#1 and anti-NC (*P* < 0.05) (Figure 5A). First, we assessed the proliferation of U2OS and MG-63 cells using an MTT assay. We found that the proliferation of U2OS and MG-63 cells transfected with si-PVT1#1 was decreased compared with the proliferation of control cells, whereas the proliferation of U2OS and MG-63 cells transfected with si-PVT1#1 and anti-miR-195 was increased compared with cells transfected with si-PVT1#1 and anti-NC (Figure 5B–5C). Similarly, we assessed the proliferation of U2OS and MG-63 cells using clonal colony-forming assay, and the result of this was similar to that of the MTT assay (Figure 5D). Second, we analyzed cell apoptosis with Annexin-V/7-AAD staining, which indicated that apoptosis was increased in U2OS and MG-63 cells transfected with si-PVT1#1 compared with control cells, whereas apoptosis was decreased in U2OS and MG-63 cells transfected with si-PVT1#1 and anti-miR-195 compared with cells transfected si-PVT1#1 and anti-NC (Figure 5E). We then assessed migration and invasion using a Transwell assay. Our results revealed that the migration and invasion of U2OS and MG-63 cells transfected with si-PVT1#1 were decreased compared with those of control cells, whereas the migration and invasion of U2OS and MG-63 cells transfected with si-PVT1#1 and anti-miR-195 were increased compared with those of cells transfected with si-PVT1#1 and anti-NC (Figure 5F–5G). Furthermore, we found a significant increase in the number of G1-phase cells and a significant decrease in the number of S-phase cells in U2OS transfected with si-PVT1#1 compared with control cells, whereas the number of G1-phase cells significantly decreased and the number of S-phase cells significantly increased in U2OS transfected with si-PVT1#1 and anti-miR-195 compared with cells transfected with si-PVT1#1 and anti-NC (Figure 5H). A similar result was found in MG-63 cells (Figure 5I). Therefore, silencing PVT1 by siRNA suppresses proliferation, migration and invasion and promotes cell cycle arrest and apoptosis via miR-195 in osteosarcoma cells.

### Silencing PVT1 by siRNA suppresses BCL2, CCND1, and FASN protein expression via miR-195 in osteosarcoma cells

**Figure 6:**
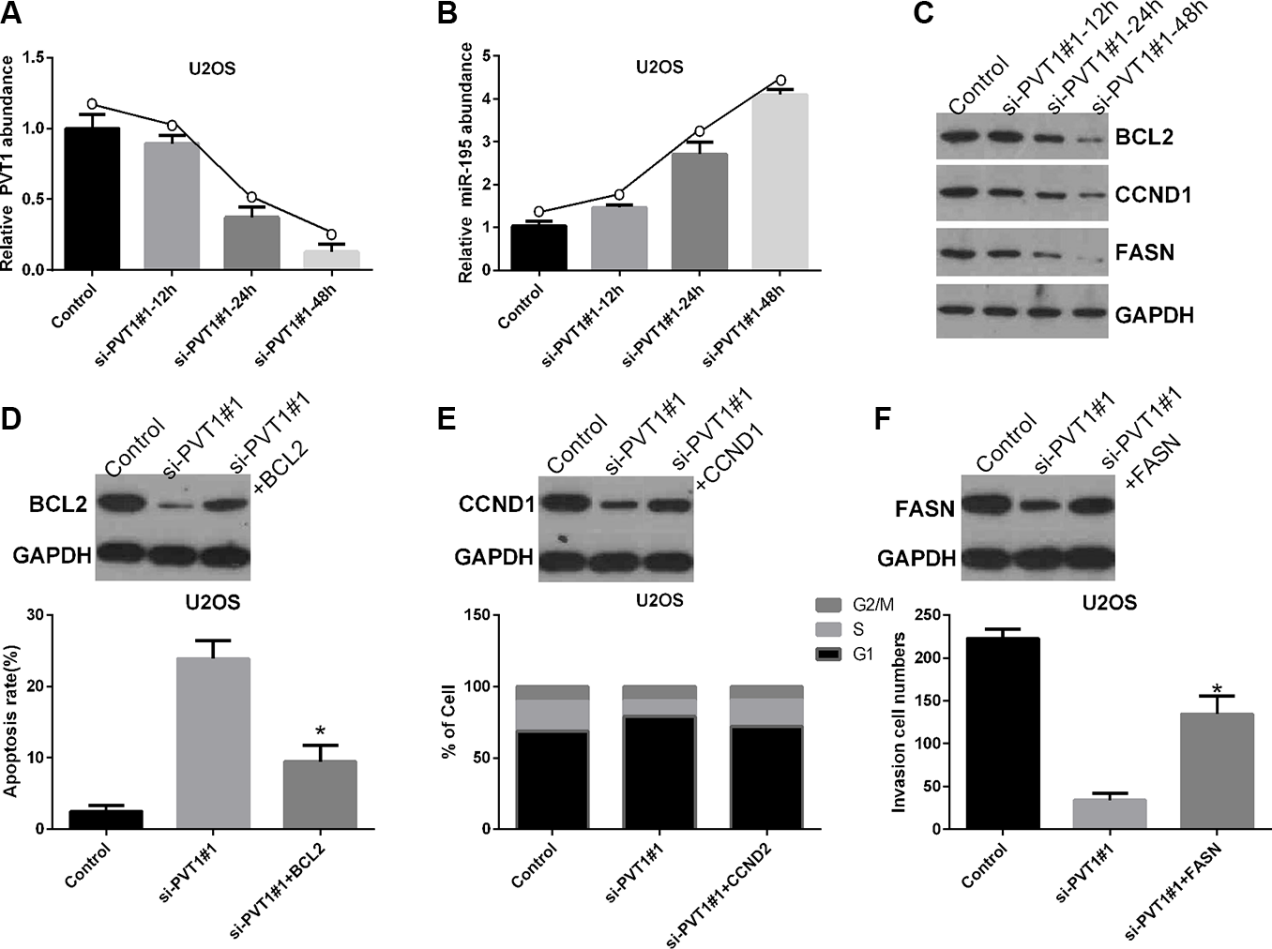
Silencing PVT1 by siRNA suppresses BCL2, CCND1, and FASN protein expression via miR-195 in osteosarcoma cells (**A**) U2OS cells were transfected with control or si-PVT1#1 for 12, 24, and 48 hrs. The PVT1 mRNA level was measured by qRT-PCR (^*^P < 0.05). (**B**) The miR-195 mRNA level was measured by qRT-PCR in U2OS cells treated as described in A (*P < 0.05). (**C**) Western blotting was used to measure the protein expression levels of BCL2, CCND1, and FASN in U2OS cells treated as described in A; GAPDH was used as a protein-loading control. (**D**) BCL2 inhibited si-PVT1#1-induced apoptosis in U2OS cells (^*^P < 0.05). (**E**) CCND1 inhibited si-PVT1#1-mediated cell cycle arrest in U2OS cells (^*^P < 0.05). (**F**) FASN attenuated the si-PVT1#1-mediated inhibition of U2OS cell invasion (^*^P < 0.05).

To study the role of miR-195 in the mechanisms and functions of PVT1, we analyzed direct targets of miR-195 using oncomiRDB and found that BCL2, CCND1, and FASN may be related to miR-195, as shown in Table 2. Therefore, U2OS cells were transfected with control and si-PVT1#1 for 12, 24, or 48 hrs. We found transfection with si-PVT1#1 for 12, 24, and 48 hrs gradually decreased the level of PVT1 mRNA in U2OS cells compared with control cells (Figure 6A), whereas the level of miR-195 mRNA gradually increased (Figure 6B). Moreover, the protein expression levels of BCL2, CCND1, and FASN gradually decreased in U2OS cells transfected with si-PVT1#1 for 12, 24, and 48 hrs compared with control cells (Figure 6C). Furthermore, our results revealed that BCL2 inhibited si-PVT1#1-induced apoptosis in U2OS cells (*P* < 0.05) (Figure 6D). Moreover, CCND1 inhibited si-PVT1#1-induced cell cycle arrest in U2OS cells (*P* < 0.05) (Figure 6E), and FASN attenuated the si-PVT1#1-mediated inhibition of U2OS cell invasion (*P* < 0.05) (Figure 6F).

**Table 2:** miR-195 directly targets summered by oncomiRDB

pubmedID	tissue	miRNAs	direct_targets	miRNA_function	sum_effect
23162665	bone and muscle	miR-195	FASN(2194)	decrease cell migration decrease cell invasion	tumor-suppressive
22888524	liver	miR-195	LATS2(26524)	increase apoptosis	tumor-suppressive
22289176	bladder	miR-195	CDK4(1019)	induce cell cycle G1 arrest inhibit cell growth	tumor-suppressive
22217655	brain	miR-195	NPA	inhibit cell cycle progression inhibit cell invasion	tumor-suppressive
21947305	liver	miR-195	BCL2L2(599)	reduce 5-FU resistance	NPA
21350001	breast	miR-195	CCND1(595) RAF1(5894)	reduce cell proliferation reduce cell invasion	tumor-suppressive
20727858	colorectum	miR-195	BCL2(596)	reduce cell viability promote cell apoptosis suppress tumorigenicity	tumor-suppressive
19441017	liver	miR-195	CCND1(595) CDK6(1021) E2F3(1871)	inhibit cell cycle G1/S transition inhibit colony formation	tumor-suppressive

### PVT1 exhibits oncogenic activity in osteosarcoma *in vivo*

**Figure 7:**
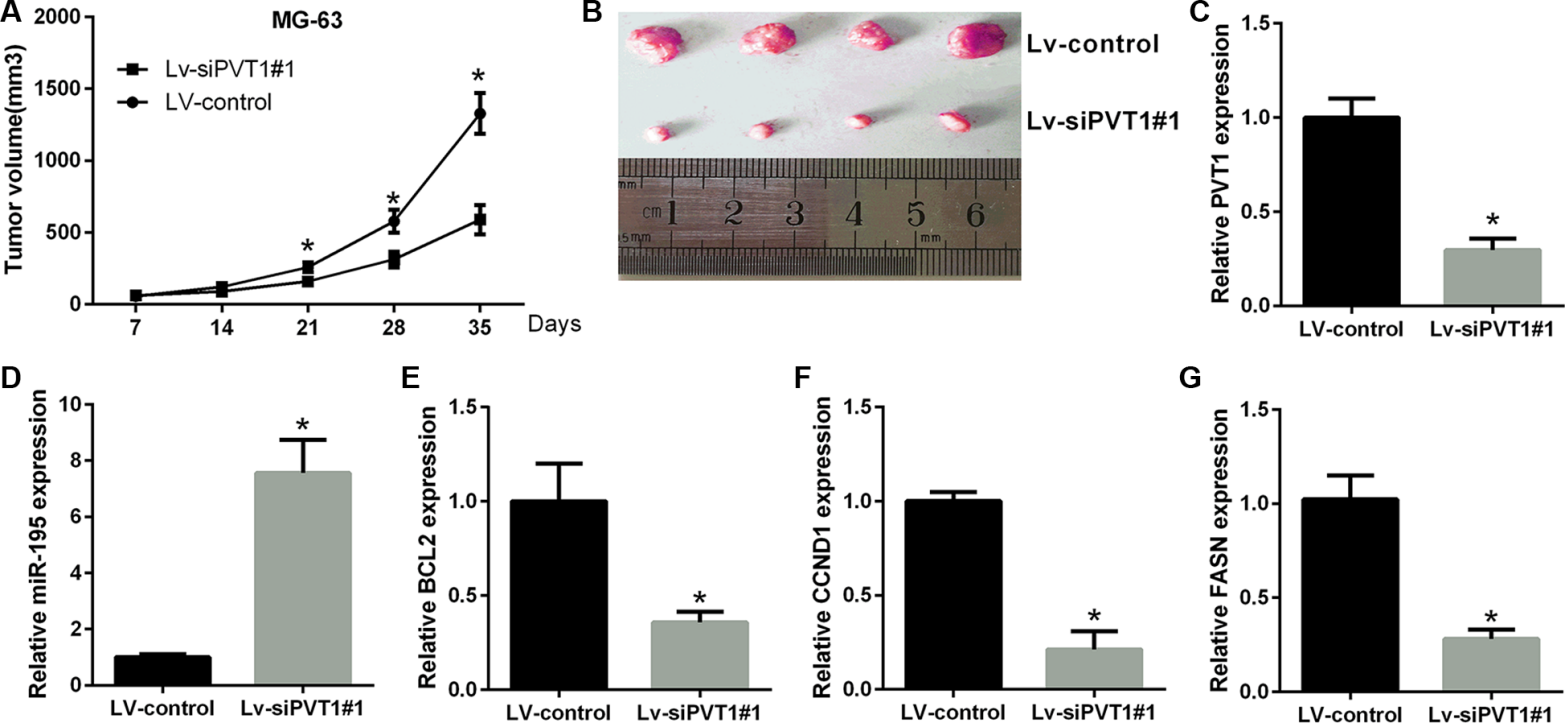
PVT1 promotes tumor growth *in vivo* (**A**) Statistical analysis of changes in tumor volume *in vivo*. (**B**) Representative image of tumor formation in nude mice 35 days after the subcutaneous administration of Lv-shRNA-PVT1#1 and Lv-control cells. (**C**–**G**) The results of qRT-PCR analyses for PVT1, miR-195, BCL2, CCND1 and FASN after PVT1 silencing *in vivo* (^**^P < 0.05).

To further test the oncogenic activity of PVT1, MG-63 cells stably expressing control shRNA or sh-PVT1#1 were injected subcutaneously into nude mice. The tumor volumes were measured weekly (7 days), and the mice were sacrificed after five weeks (35 days). Compared to the MG-63-vector group, tumor growth was reduced in the si-PVT1#1 group (Figure 7A and 7B). Furthermore, in agreement with *in vitro* results, the qRT-PCR data demonstrate that the levels of PVT1, BCL2, CCND1 and FASN were lower, whereas those of miR-195 were higher in the siPVT1#1 group than in the control group (Figure 7C–7G). This finding suggests that the oncogenic role of PVT1 is mediated by miR-195 in osteosarcoma tumors *in vivo*.

### The gene network of PVT1 in osteosarcoma

**Figure 8:**
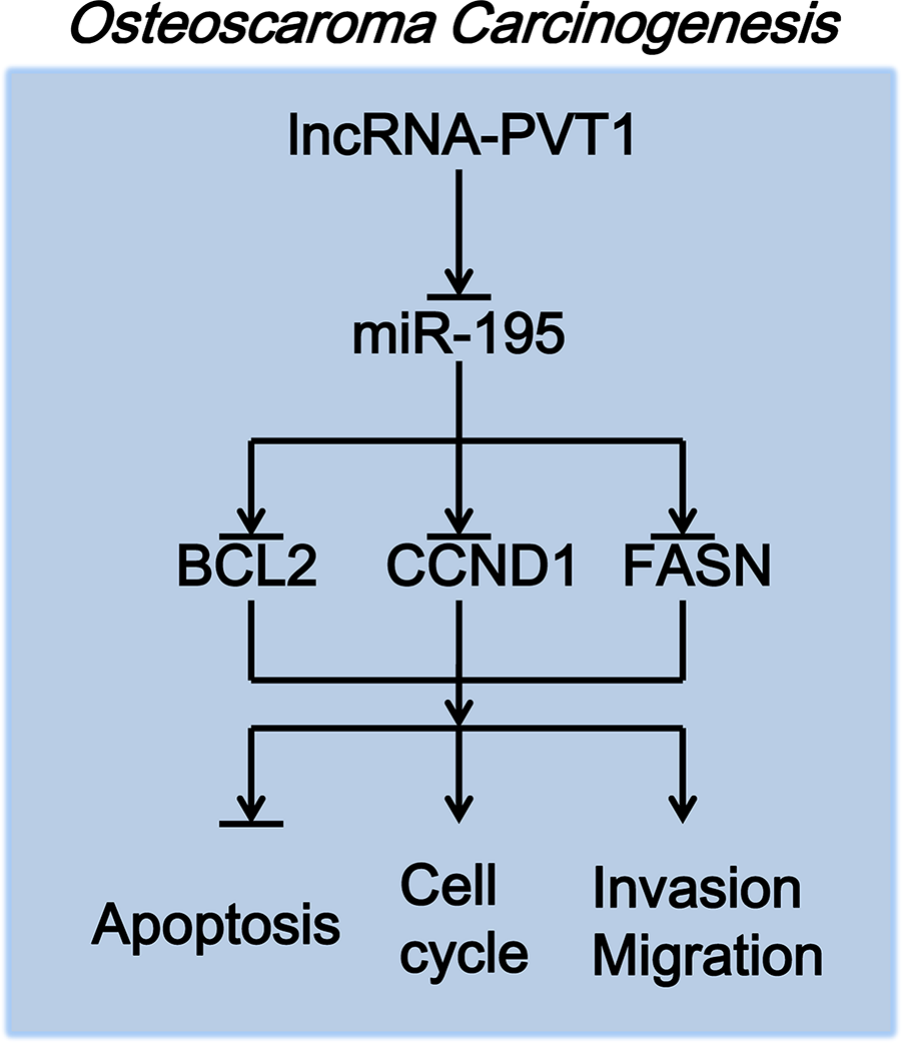
The molecular mechanism of PVT1 action in osteosarcoma carcinogenesis

PVT1 increased the expression levels of BCL2, CCND1 and FASN by inhibiting miR-195 in osteosarcoma. First, lncRNA-PVT1 inhibits the expression of miR-195, and miR-195 then inhibits the expression of BCL2, CCND1 and FASN. PVT1 consequently inhibits the apoptosis of osteosarcoma cells via miR-195/BCL2, induces cell-cycle arrest in osteosarcoma cells via miR-195/CCND1, and promotes the migration and invasion of osteosarcoma cells via miR-195/FASN (Figure 8).

## DISCUSSION

LncRNAs can regulate protein-coding genes, transcription, and post-transcription, and they play important roles in biological processes [30, 31]. Many studies have shown that lncRNAs act as oncogenes or cancer suppressor genes to affect tumorigenesis, metastasis, prognosis or diagnosis [30, 32]. Specifically, PVT-1 occupies a unique cell context within the 8q24.21 region and was demonstrated to be required for the high MYC protein levels in 8q24-amplified human cancer cells [33]. The mechanism and function of PVT1 have been studied in various cancers. For example, lncRNA PVT1 accelerated tumorigenesis of non-small cell lung cancer [34] and was associated with a poor prognosis in patients with pancreatic cancer [35]. Moreover, PVT1 promoted proliferation and was associated with a poor prognosis in gastric cancer [36], and PVT-1 resulted in a poor prognosis by inhibiting apoptosis of colorectal cancers [37]. Furthermore, PVT1 participated in tumor progression of hepatocellular carcinoma patients [38]. In our study, PVT1 expression was higher in osteosarcoma tissues than corresponding noncancerous tissues. Concurrently, PVT1 was associated with reduced survival in osteosarcoma patients. We also proved that PVT1 expression was higher in metastatic osteosarcoma tissues than in primary osteosarcoma tissues. Furthermore, we demonstrated that silencing PVT1 inhibited proliferation, migration and invasion and promoted apoptosis and cell cycle arrest in osteosarcoma cells. Therefore, we speculated that PVT1 may play a vital role in the development and progression of osteosarcoma.

An increasing number of studies have shown that miRNAs play important roles in regulating biological processes, including cell proliferation, metastasis, apoptosis, angiogenesis, and inflammation, in a variety of tumors [39, 40]. Specifically, many studies have indicated that various miRNAs, such as miRNA-143, miRNA199a-3p, and miRNA-21, were related to the development of osteosarcoma [41–43]. For example, lncRNAs can act as ceRNA sponges for miRNAs to regulate the degradation of miRNA targets. In our study, we selected 7 miRNAs associated with PVT1, miR-365, miR-27a, miR-141, miR-17-5p, miR-455, and miR-195, using a gene microarray. We further validated the mRNA expression levels of miRNAs and found that miR-195 was highly expressed in MG-63 cells. We then found that PVT1 negatively regulated miR-195 in osteosarcoma cells, and silencing PVT1 suppressed proliferation, migration and invasion and promoted cell cycle arrest and apoptosis via miR-195 in osteosarcoma cells.

Apoptosis is the process of genetically programmed cell death, which is conserved and highly regulated at the molecular level and participates in embryonic development and the destruction of diseased or damaged cells. Cells may be driven to apoptosis via the death receptor or mitochondrial pathway. Mitochondrial outer membrane permeabilization (MOMP) may release cell death factors, such as cytochrome c, into the cytoplasm, which activates caspase and destroys essential cellular components [44]. The B-cell lymphoma 2 protein (BCL-2) family, including BCL-2, BCL-W, BCL-XL, A1 (BCL-2 related protein A1) and MCL-1 (myeloid cell leukemia sequence 1 protein), can induce MOMP. Accordingly, we found that BCL2 inhibited si-PVT1#1-apoptosis in U2OS cells.

Cyclin D1 (CCND1) plays an important role in regulating the progression from the G1-phase to the S-phase by forming complexes in the cytoplasm that will enter the nucleus and destroy cell-cycle suppressive retinoblastoma protein [45]. A previous study has indicated that miR-15a and miR-16-1 inhibit apoptosis and cell cycle arrest by downregulating CCND1 in osteosarcoma [46]. In our study, CCND1 inhibited si-PVT1#1-induced cell cycle arrest in U2OS cells.

Fatty acid synthase (FASN) is a key enzyme for endogenous lipogenesis that can promote the synthesis of long-chain fatty acids [47], and recent studies have shown that FASN may be associated with the metastasis of cancer cells [48]. For example, miRNA-195 suppressed the proliferation, invasion and metastasis of breast cancer cells via FASN, HMGCR, ACACA and CYP27B1 [49]. Moreover, miRNA-195 inhibited the migration and invasion of osteosarcoma cells via FASN [47]. Accordingly, o results showed that FASN attenuated the si-PVT1#1-mediated inhibition of U2OS cell invasion.

In summary, our results indicated that lncRNA PVT1 was overexpressed in osteosarcoma and decreased the survival rate of osteosarcoma patients. Moreover, silencing PVT1 by siRNA inhibited proliferation, migration and invasion and promoted apoptosis and cell cycle arrest in osteosarcoma cells via miR-195. A gene microarray was then used to screen differentially expressed miRNAs associated with PVT1. Specifically, PVT1 negatively regulated miR-195 in osteosarcoma cells, and silencing PVT1 by siRNA suppressed BCL2, CCND1, and FASN protein expression via miR-195 in osteosarcoma cells. Our study demonstrated that PVT1 may be a therapeutic target for the treatment of osteosarcoma.

## MATERIALS AND METHODS

### Clinical specimens

In this study, osteosarcoma tissues and corresponding noncancerous tissues (at least 5 cm from the edge of the cancer) were collected from 26 patients who had received surgery at three hospitals (Huai'an Hospital Affiliated of Xuzhou Medical College and Huai'an Second Hospital, Huai'an First People's Hospital and Second People's Hospital of Hunan Province) from 2014 to 2015. Written informed consent was obtained from all patients, and the study of clinical specimens was approved by the Ethics Committees of the three hospitals. The histological diagnosis of osteosarcoma was assessed following the World Health Organization (WHO). All tissue specimens were stored at −80°C until use.

### Cell lines

The human normal osteoblast cell line NHost and several osteosarcoma cell lines (KHOS, 143b, LM7, U2OS, and MG-63) were obtained from the Type Culture Collection of the Chinese Academy of Sciences, Shanghai, China. All cells were cultured in Dulbecco's modified Eagle medium (DMEM) (Invitrogen, Carlsbad, CA, USA) containing 10% fetal bovine serum (FBS; Invitrogen, Carlsbad, CA, USA) penicillin (100 U/ml), and streptomycin (100 μg/ml) at 37°C and 5% CO_2_.

### RNA interference

For the treatment, siRNAs against PVT1 and negative control siRNA were purchased from GenePharma (GenePharma Co., Ltd., Shanghai, China). The sequences of selected regions to be targeted by siRNAs for PVT1 were [37] PVT-1 siRNA-1#: 5′-GCU UGG AGG CUG AGG AGU UTT-3′, PVT-1 siRNA-2#: 5′-CCC AAC AGG AGG ACA GCU UTT-3′, and PVT-1 siRNA-3#: 5′-CAG CCA UCA UGA UGG UAC U-3′. U2OS and MG-63 cells (2 × 10^5^ cells/well) were cultured in 6-well plates and then transfected with 50 nM siRNA (Negative control, NC), siRNA-1#, siRNA-2#, or siRNA-3# using Lipofectamine 3000 (Invitrogen, Carlsbad, CA, USA) according to the manufacturer's instructions. Similarly, cells were transfected with 50 nmol/L negative control, or anti-miR-195 oligonucleotides (GenePharma, Shanghai, China).

### Quantitative real-time reverse transcription PCR (qRT-PCR)

According to the manufacturer's instructions, total RNA was extracted from tissue samples or cells using TRIzol reagent (Invitrogen, CA, USA). The reverse transcription (RT) reaction was performed using the RevertAid First Strand cDNA Synthesis Kit (Thermo Fisher Scientific Inc., Rockford, IL, USA). The RT primer for miR-195 was 5′-TGT CAG GCA ACC GTA TTC ACC GGA GTG GTG GGA AG-3′; the RT primer for U6 was 5′-CGC TTC ACG AAT TTG CGT GTC AT-3′. As described previously [50], the mRNA expression levels of genes were evaluated using the SYBR-Green PCR Master Mix kit (Takala, Dalian, China) and an ABI7500 Real-time PCR system (Applied Biosystems). The primer sequences for GAPDH are: 5′-TGT TCG TCAT GGG TGT GAA C-3′ (the forward primer) and 5′-ATG GCA TGG ACT GTG GTC AT-3′ (the reverse primer) (internal control); the primer sequences for PVT1 are: 5′-TGA GAA CTG TCC TTA CGT GAC C-3′ (the forward primer) and 5′-AGA GCA CCA AGA CTG GCT CT-3′ (the reverse primer); the primer sequences for U6 are: 5′-CTC GCT TCG GCA GCA CA-3′ (the forward primer) and 5′-AAC GCT TCA CGA ATT TGC GT-3′ (the reverse primer); the primer sequences for miR-195 are: 5′-GAA TTC GCC TCA AGA GAA CAA AGT GGA G-3′ (the forward primer) and 5′- AGA TCT CCC ATG GGG GCT CAG CCC CT -3′ (the reverse primer). The mRNA expression level of miR-195 was normalized to that of U6; the mRNA expression level of PVT1 was normalized to that of Glyceraldehyde 3-phosphate dehydrogenase (GAPDH).

### MiRNA microarray analysis

According to previous research [51, 52], the mirVana microRNA Labeling kit (Ambion, Austin, TX, USA) was used to label miRNAs. Monoreactive Cy3 dye (Amersham Pharmacia Biotech Ltd, Saclay, France) was used for labeling. The fluorescent probes were lyophilized, and the slides were then washed and scanned by a Generation III array scanner (Amersham Pharmacia, Piscataway, NJ, USA).

### Chromatin immunoprecipitation (ChIP) assays

ChIP assays were performed using a CHIP assay kit (Millipore, Billerica, MA). According to the manufacturer's instructions, the cultured U2OS and MG-63 cells were fixed with a final concentration of 1% formaldehyde. Pelleted cells were resuspended in SDS lysis buffer and sonicated to shear DNA to lengths between 200–1000 bp according to the manufacturer's instructions. Ten microliters of supernatant were used as input, and the remaining lysate diluted with ChIP dilution buffer containing protease inhibitor. Subsequently, 60 μL of a protein agarose/salmon sperm DNA (50% slurry) was then added to the chromatin solution, which was then immunoprecipitated with PVT1 antibody and IgG (Abcam). The agarose/antibody/histone complex was washed with complex wash buffer, and 20 μL of 5 M NaCl was then added to the immune complexes. Histone-DNA crosslinks were reversed by heating at 65°C for 4 hours. The samples were treated with proteinase K (Promega) for 1 h at 45°C, and glycogen (Solarbo, Beijing, China) was added. DNA was extracted with 25:24:1 Phenol/chloroform/isoamyl alcohol, and purified DNA was then analyzed by PCR.

### Luciferase reporter assays

The treated U2OS and MG-63 cells (5 × 10^4^ cells/well) were cultured in 24-well plates and co-transfected with pcDNA3.1 containing the wild type (PVT1-WT) or mutant (PVT1-Mut) and pcDNA3.1-miR-195 using Lipofectamine 3000 (Invitrogen); a renilla plasmid (RL-SV40) was also transfected as an internal control. According to the manufacturer's instructions, the luciferase activities were measured using a dual-luciferase reporter assay system (Promega, Madison, WI).

### Western blot analysis

Cells were lysed using Radio Immunoprecipitation Assay (RIPA) buffer (Thermo Scientific, Rockford, IL, USA) containing a protease inhibitor cocktail (P8340; Sigma-Aldrich, St. Louis, MO, USA), and 30 μg of protein was then separated on an 8% sodium dodecyl sulfate polyacrylamide (SDS/PAGE) gel. The proteins were then transferred to a polyvinylidene fluoride membrane (PVDF, Millipore, Billerica, MA), which was blocked for 2 hrs with 5% skim milk at room temperature and incubated with primary antibodies at 4°C overnight. The next day, the PVDF membrane was incubated with horseradish peroxidase (HRP) secondary antibody for 1 h at room temperature, and protein expression was then detected using the enhanced chemiluminescence (ECL) substrate kit (Amersham Biosciences, Inc.) and the enhanced chemiluminescence detection system (Amersham Biosciences, Piscataway, New Jersey, USA). The antibodies used in this study included anti-GAPDH antibody (1: 2000, Santa Cruz Biotechnology, Santa Cruz, CA, USA), anti-BCL2 antibody (1: 250, Santa Cruz Biotechnology), anti-CCND1 antibody (1: 200, Abcam Company, Cambridge, MA, USA), and anti-FASN antibody (1: 100, Abcam Company, Cambridge, MA, USA).

### Colony-forming unit (CFU) assay

The treated U2OS and MG-63 cells were cultured DMEM complete medium for 14 days, and the colonies were then fixed with methanol and dyed with Giemsa dye solution before counting the colony-forming units.

### MTT assay

The treated U2OS and MG-63 cells (2000 cells/well) were seeded in 96-well plates for 0, 12, 24, 48 or 72 hrs. An MTT (5 mg/mL, 20 μl/well) assay was used to measure cell viability. After 4 hrs, 100 μl of dimethyl sulfoxide solution (DMSO) was used to dissolve the formazan crystals, and the absorbance was measured at 570 nm using a micro-plate reader (Bio Tek Instruments, Inc., Winooski, VT, USA)

### Migration and invasion assays

Briefly, 200 μl of treated U2OS and MG-63 cells (5 × 10^5^ cells/well) incubated in serum-free medium were seeded in the upper chamber of a Transwell (Corning Costar Corp., Cambridge, MA, USA), and the lower chamber was filled with complete medium before allowing the cells to migrate for 24 hrs. Migratory cells were fixed with paraformaldehyde and stained with 0.1% crystal violet solution. Five fields were counted using a 20× objective. For the invasion assays, the upper chambers were pre-coated with Matrigel (BD Biosciences, San Diego, CA, USA).

### Flow cytometric analysis of the cell cycle

The treated U2OS and MG-63 cells were resuspended using PBS containing 70% ethanol, RNaseA (0.5 mg/ml) and propidium iodide (0.1 mg/ml). FACS Calibur (BD Biosciences) was used to obtain images of the cell cycle, and the Flowjo software (Tree Star Corp, Ashland, OR) was used to analyze the results.

### Flow cytometric analysis of the cell apoptosis

The treated U2OS and MG-63 cells were resuspended and stained with Annexin-V and 7-AAD. Similarly, FACS Calibur was used to obtain images of cell apoptosis, and flow cytometry was used to analyze the results.

### Statistical analysis

The results were analyzed using GraphPad Prism (GraphPad Software Inc, La Jolla, CA) and the SPSS 15.0 software (SPSS, Chicago, IL, USA). The significance of differences was assessed using Student's *t*-test or a one-way ANOVA. All data are expressed as the means ± SDs. *P* < 0.05 indicates a significant difference.

## References

[1] Ando K, Heymann MF, Stresing V, Mori K, Redini F, Heymann D (2013). Current therapeutic strategies and novel approaches in osteosarcoma. Cancers (Basel).

[2] Dorfman HD, Czerniak B (1995). Bone cancers. Cancer.

[3] Stiller CA (2007). International patterns of cancer incidence in adolescents. Cancer Treat Rev.

[4] Yang J, Zhang W (2013). New molecular insights into osteosarcoma targeted therapy. Curr Opin Oncol.

[5] Marcove RC, Mike V, Hajek JV, Levin AG, Hutter RV (1970). Osteogenic sarcoma under the age of twenty-one. A review of one hundred and forty-five operative cases. J Bone Joint Surg Am.

[6] Liu X, Li D, Zhang W, Guo M, Zhan Q (2012). Long non-coding RNA gadd7 interacts with TDP-43 and regulates Cdk6 mRNA decay. EMBO J.

[7] Lakhotia SC (2012). Long non-coding RNAs coordinate cellular responses to stress. Wiley Interdiscip Rev RNA.

[8] Paralkar VR, Weiss MJ (2011). A new 'Linc' between noncoding RNAs and blood development. Genes Dev.

[9] Li CH, Chen Y (2013). Targeting long non-coding RNAs in cancers: progress and prospects. Int J Biochem Cell Biol.

[10] Audas TE, Lee S (2016). Stressing out over long noncoding RNA. Biochim Biophys Acta.

[11] Yang F, Zhang L, Huo XS, Yuan JH, Xu D, Yuan SX, Zhu N, Zhou WP, Yang GS, Wang YZ, Shang JL, Gao CF, Zhang FR (2011). Long noncoding RNA high expression in hepatocellular carcinoma facilitates tumor growth through enhancer of zeste homolog 2 in humans. Hepatology.

[12] Enfield KS, Pikor LA, Martinez VD, Lam WL (2012). Mechanistic Roles of Noncoding RNAs in Lung Cancer Biology and Their Clinical Implications. Genet Res Int.

[13] Piao HL, Ma L (2012). Non-coding RNAs as regulators of mammary development and breast cancer. J Mammary Gland Biol Neoplasia.

[14] Zhao W, An Y, Liang Y, Xie XW (2014). Role of HOTAIR long noncoding RNA in metastatic progression of lung cancer. Eur Rev Med Pharmacol Sci.

[15] Mercer TR, Dinger ME, Mattick JS (2009). Long non-coding RNAs: insights into functions. Nat Rev Genet.

[16] Hayes EL, Lewis-Wambi JS (2015). Mechanisms of endocrine resistance in breast cancer: an overview of the proposed roles of noncoding RNA. Breast Cancer Res.

[17] Baer C, Claus R, Plass C (2013). Genome-wide epigenetic regulation of miRNAs in cancer. Cancer Res.

[18] Pimentel AM, Kobayashi D, Kliemann LM, Franjdlich R, Capp E, Corleta HV (2012). Transvaginal ultrasound ovarian diathermy: sheep as an experimental model. J Ovarian Res.

[19] Farazi TA, Hoell JI, Morozov P, Tuschl T (2013). MicroRNAs in human cancer. Adv Exp Med Biol.

[20] Di Leva G, Croce CM (2013). The Role of microRNAs in the Tumorigenesis of Ovarian Cancer. Front Oncol.

[21] Garzon R, Calin GA, Croce CM (2009). MicroRNAs in Cancer. Annu Rev Med.

[22] Liz J, Esteller M (2016). lncRNAs and microRNAs with a role in cancer development. Biochim Biophys Acta.

[23] Tay Y, Rinn J, Pandolfi PP (2014). The multilayered complexity of ceRNA crosstalk and competition. Nature.

[24] Karreth FA, Pandolfi PP (2013). ceRNA cross-talk in cancer: when ce-bling rivalries go awry. Cancer Discov.

[25] Song X, Cao G, Jing L, Lin S, Wang X, Zhang J, Wang M, Liu W, Lv C (2014). Analysing the relationship between lncRNA and protein-coding gene and the role of lncRNA as ceRNA in pulmonary fibrosis. J Cell Mol Med.

[26] Liang WC, Fu WM, Wong CW, Wang Y, Wang WM, Hu GX, Zhang L, Xiao LJ, Wan DC, Zhang JF, Waye MM (2015). The lncRNA H19 promotes epithelial to mesenchymal transition by functioning as miRNA sponges in colorectal cancer. Oncotarget.

[27] Zhou X, Ye F, Yin C, Zhuang Y, Yue G, Zhang G (2015). The Interaction Between MiR-141 and lncRNA-H19 in Regulating Cell Proliferation and Migration in Gastric Cancer. Cell Physiol Biochem.

[28] Peng W, Si S, Zhang Q, Li C, Zhao F, Wang F, Yu J, Ma R (2015). Long non-coding RNA MEG3 functions as a competing endogenous RNA to regulate gastric cancer progression. J Exp Clin Cancer Res.

[29] Doonan F, Cotter TG (2013). Detection of DNA fragmentation in retinal apoptosis by TUNEL. Methods Mol Biol.

[30] Qiu MT, Hu JW, Yin R, Xu L (2013). Long noncoding RNA: an emerging paradigm of cancer research. Tumour Biol.

[31] Eades G, Zhang YS, Li QL, Xia JX, Yao Y, Zhou Q (2014). Long non-coding RNAs in stem cells and cancer. World J Clin Oncol.

[32] He Y, Meng XM, Huang C, Wu BM, Zhang L, Lv XW, Li J (2014). Long noncoding RNAs: Novel insights into hepatocelluar carcinoma. Cancer Lett.

[33] Tseng YY, Moriarity BS, Gong W, Akiyama R, Tiwari A, Kawakami H, Ronning P, Reuland B, Guenther K, Beadnell TC, Essig J, Otto GM, O'Sullivan MG (2014). PVT1 dependence in cancer with MYC copy-number increase. Nature.

[34] Yang YR, Zang SZ, Zhong CL, Li YX, Zhao SS, Feng XJ (2014). Increased expression of the lncRNA PVT1 promotes tumorigenesis in non-small cell lung cancer. Int J Clin Exp Pathol.

[35] Huang C, Yu W, Wang Q, Cui H, Wang Y, Zhang L, Han F, Huang T (2015). Increased expression of the lncRNA PVT1 is associated with poor prognosis in pancreatic cancer patients. Minerva Med.

[36] Kodet O, Lacina L, Krejci E, Dvorankova B, Grim M, Stork J, Kodetova D, Vlcek C, Sachova J, Kolar M, Strnad H, Smetana K (2015). Melanoma cells influence the differentiation pattern of human epidermal keratinocytes. Mol Cancer.

[37] Takahashi Y, Sawada G, Kurashige J, Uchi R, Matsumura T, Ueo H, Takano Y, Eguchi H, Sudo T, Sugimachi K, Yamamoto H, Doki Y, Mori M (2014). Amplification of PVT-1 is involved in poor prognosis via apoptosis inhibition in colorectal cancers. Br J Cancer.

[38] Ding C, Yang Z, Lv Z, Du C, Xiao H, Peng C, Cheng S, Xie H, Zhou L, Wu J, Zheng S (2015). Long non-coding RNA PVT1 is associated with tumor progression and predicts recurrence in hepatocellular carcinoma patients. Oncol Lett.

[39] Wang J, Paris PL, Chen J, Ngo V, Yao H, Frazier ML, Killary AM, Liu CG, Liang H, Mathy C, Bondada S, Kirkwood K, Sen S (2015). Next generation sequencing of pancreatic cyst fluid microRNAs from low grade-benign and high grade-invasive lesions. Cancer Lett.

[40] Stahlhut C, Slack FJ (2013). MicroRNAs and the cancer phenotype: profiling, signatures and clinical implications. Genome Med.

[41] Osaki M, Takeshita F, Sugimoto Y, Kosaka N, Yamamoto Y, Yoshioka Y, Kobayashi E, Yamada T, Kawai A, Inoue T, Ito H, Oshimura M, Ochiya T (2011). MicroRNA-143 regulates human osteosarcoma metastasis by regulating matrix metalloprotease-13 expression. Mol Ther.

[42] Duan Z, Choy E, Harmon D, Liu X, Susa M, Mankin H, Hornicek F (2011). MicroRNA-199a-3p is downregulated in human osteosarcoma and regulates cell proliferation and migration. Mol Cancer Ther.

[43] Ziyan W, Shuhua Y, Xiufang W, Xiaoyun L (2011). MicroRNA-21 is involved in osteosarcoma cell invasion and migration. Med Oncol.

[44] Roy MJ, Vom A, Czabotar PE, Lessene G (2014). Cell death and the mitochondria: therapeutic targeting of the BCL-2 family-driven pathway. Br J Pharmacol.

[45] Diehl JA (2002). Cycling to cancer with cyclin D1. Cancer Biol Ther.

[46] Cai CK, Zhao GY, Tian LY, Liu L, Yan K, Ma YL, Ji ZW, Li XX, Han K, Gao J, Qiu XC, Fan QY, Yang TT (2012). miR-15a and miR-16-1 downregulate CCND1 and induce apoptosis and cell cycle arrest in osteosarcoma. Oncol Rep.

[47] Mao JH, Zhou RP, Peng AF, Liu ZL, Huang SH, Long XH, Shu Y (2012). microRNA-195 suppresses osteosarcoma cell invasion and migration in vitro by targeting FASN. Oncol Lett.

[48] Murata S, Yanagisawa K, Fukunaga K, Oda T, Kobayashi A, Sasaki R, Ohkohchi N (2010). Fatty acid synthase inhibitor cerulenin suppresses liver metastasis of colon cancer in mice. Cancer Sci.

[49] Singh R, Yadav V, Kumar S, Saini N (2015). MicroRNA-195 inhibits proliferation, invasion and metastasis in breast cancer cells by targeting FASN, HMGCR, ACACA and CYP27B1. Sci Rep.

[50] Jiang L, Lai YK, Zhang J, Wang H, Lin MC, He ML, Kung HF (2011). Targeting S100P inhibits colon cancer growth and metastasis by Lentivirus-mediated RNA interference and proteomic analysis. Mol Med.

[51] Konishi H, Ichikawa D, Komatsu S, Shiozaki A, Tsujiura M, Takeshita H, Morimura R, Nagata H, Arita T, Kawaguchi T, Hirashima S, Fujiwara H, Okamoto K (2012). Detection of gastric cancer-associated microRNAs on microRNA microarray comparing pre- and post-operative plasma. Br J Cancer.

[52] Zhao JJ, Yang J, Lin J, Yao N, Zhu Y, Zheng J, Xu J, Cheng JQ, Lin JY, Ma X (2009). Identification of miRNAs associated with tumorigenesis of retinoblastoma by miRNA microarray analysis. Childs Nerv Syst.

